# Supplementary material for: Polydopamine Nanosphere with In-Situ Loaded Gentamicin and Its Antimicrobial Activity
Source: Molecules. 2020 Apr 30;25(9):2090. doi: 10.3390/molecules25092090 (PMC7250025; doi:10.3390/molecules25092090)
Supplement: Supplementary file 1 [file molecules-25-02090-s001.pdf]

Supplementary Materials

Article

Polydopamine Nanosphere with In-situ Loaded Gentamicin and its Antimicrobial Activity

Rahila Batul, Mrinal Bhave, Peter J. Mahon and Aimin Yu*

Department of Chemistry and Biotechnology, Faculty of Science, Engineering and Technology, Swinburne University of Technology, Hawthorn, Victoria 3122, Australia

* Correspondence: aiminyu@swin.edu.au

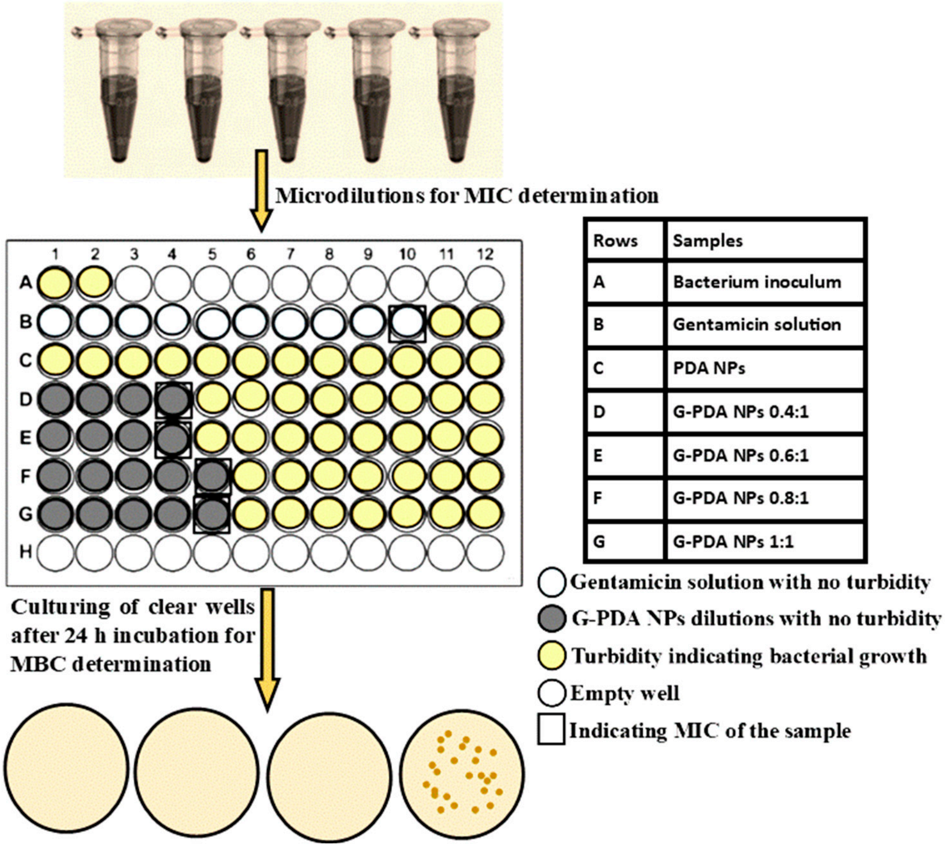

Figure S1. Schematic diagram representing method used for antimicrobial activity of G-PDA. NPs.

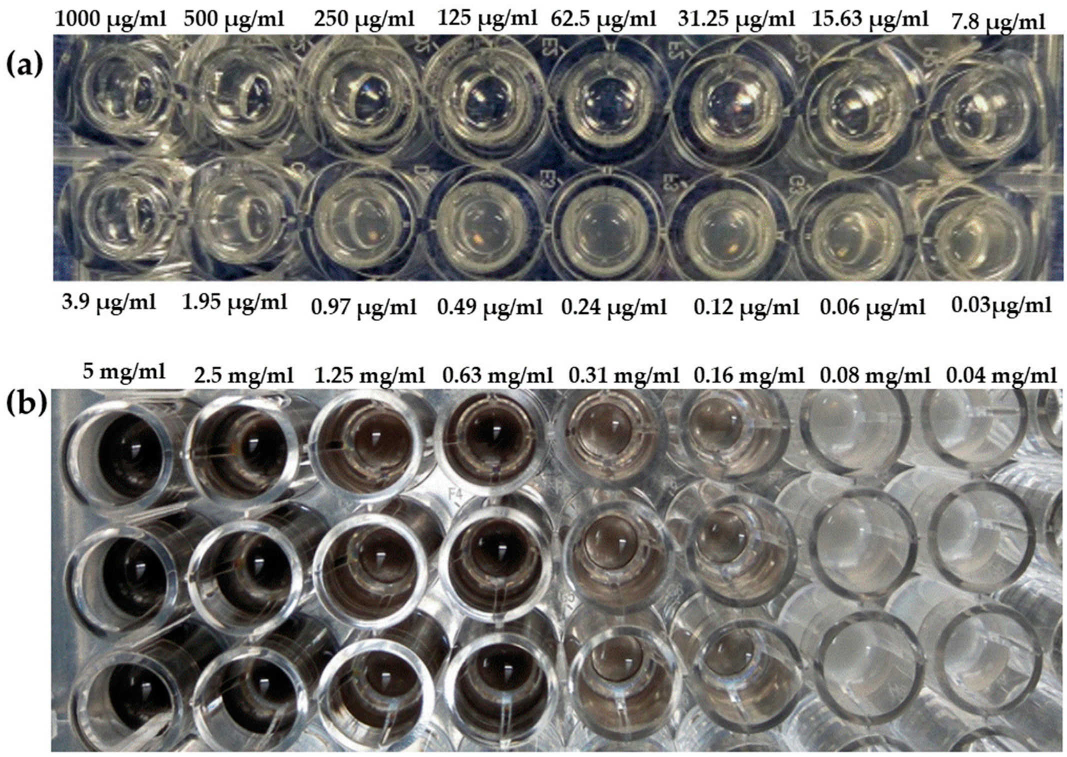

Figure S2. (**a**) Indicating MIC of gentamicin only; and (**b**) indicating one of the batch from G-PDA NPs 1:1 (in triplicate) as an example, showing visible bacterial growth after well no. 5 from left towards right. The values are presenting half serial dilutions alongside of each well.

Table S1. Atomic percentages of possible elemental states in XPS spectra of PDA and G-PDA NPs.

Main Elements.	Samples	Possible Element State	Peak Position (eV)	Relative Intensities (%)
**C1s**	PDA	C-C	284.1	51.21
		C-N	285.6	35.13
		C-O	287.2	9.73
		C=O	288.5	2.65
	G-PDA	C-C	284.0	40.55
		C-N	285.5	45.28
		C-O	287.1	12.07
		C=O	288.2	2.10
**N1s**	PDA	N-C	401.1	35.24
		C-N-C	399.5	48.80
		=N-C	398.6	15.96
	G-PDA	N-C	401.3	43.50
		C-N-C	399.6	20.15
		=N-R	398.6	37.31
**O1s**	PDA	O=C	531.5	43.51
		O-C	533.1	56.49
	G-PDA	O=C	531.6	25.76
		O-C	533.1	74.24
